# Artificial Intelligence in Inherited Epidermolysis Bullosa: Current Evidence, Challenges, and Future Directions

**DOI:** 10.3390/diagnostics16132022

**Published:** 2026-06-29

**Authors:** Ashjan Alheggi

**Affiliations:** Department of Dermatology, College of Medicine, Imam Mohammad Ibn Saud Islamic University (IMSIU), P.O. Box 7544, Riyadh 4233-13317, Saudi Arabia; aialheggi@imamu.edu.sa; Tel.: +44-7375430305

**Keywords:** artificial intelligence, epidermolysis bullosa, genomics, machine learning, deep learning, bioinformatics, dermatology

## Abstract

Epidermolysis bullosa (EB) comprises a group of rare inherited genodermatoses characterized by fragility and blistering of the skin and mucous membranes, chronic wounding, and significant morbidity including increased risk of squamous cell carcinoma in severe subtypes. Key unmet priorities include reducing diagnostic latency, establishing objective wound monitoring, enabling early detection of malignant transformation within chronic ulcerations, and developing therapies that durably modify disease progression. Artificial intelligence (AI) encompassing machine learning (ML), and deep learning (DL) is increasingly integrated into EB research and clinical practice to address these unmet needs. This structured narrative review synthesises current evidence on AI applications in EB spanning genetic diagnostics, wound assessment, inflammatory endotyping, drug repurposing, and emerging therapeutic technologies, and integrates evidence from registered clinical trials. In genomics, DL-based splicing prediction models and variant prioritisation frameworks accelerate pathogenic variant detection and reduce diagnostic latency. In wound care, convolutional neural networks-based platforms enable automated lesion segmentation and remote monitoring, while multimodal AI models predict healing trajectories and support stratification of wounds by chronicity. Computational transcriptomic analyses have identified candidate repurposing agents by reversing pathogenic gene expression signatures in EB tissue. Emerging convergence of AI with biosensors-integrated wound dressings and three-dimensional bioprinting of genetically corrected skin substitutes represents a transformative future direction. Translational barriers include limited EB-specific training datasets, algorithmic bias across diverse skin phototypes, the interpretability deficit of DL systems, and evolving regulatory frameworks for AI as a medical device. Expansion of internationally interoperable EB disease registries with standardised wound imaging protocols is identified as the single most impactful intervention to accelerate AI adoption. A minimum endpoint set for AI-assisted EB wound assessment, incorporating wound area trajectory, wound type classification, tissue composition, and paired patient-reported pain and itch scores, is proposed to standardise outcome reporting across future studies.

## 1. Introduction

Epidermolysis Bullosa (EB) encompasses a heterogeneous group of rare, inherited genodermatoses characterized by extreme fragility, chronic wounding, and blistering of the skin and mucous membranes [1]. EB is classified into four major subtypes based on the level of dermal–epidermal cleavage plane: EB simplex (EBS), junctional EB (JEB), dystrophic EB (DEB), and Kindler EB [1,2]. These disorders arise from mutations in genes that encode structural proteins essential for skin integrity [1,3]. Accurate and timely diagnosis of EB subtypes is crucial yet frequently delayed due to phenotypic variability, rarity of the condition, and the complexity of identifying causative mutations among over 1000 described variants across more than 20 genes [3,4,5]. Individuals with EB, particularly those with recessive DEB (RDEB), face a substantial burden of chronic wounds, recurrent infections, nutritional compromise, contractures, and a markedly elevated lifetime risk of aggressive squamous cell carcinoma (SCC) arising within chronic ulcerations, a complication responsible for the majority of disease-related mortality in adulthood [6]. Despite recent advances in EB molecular diagnostics and the regulatory approval of gene therapy for RDEB, substantial unmet clinical needs persist across the full spectrum of EB management, including effective disease-modifying interventions for chronic wound care and SCC prevention, and the identification of novel molecular targets capable of enhancing diagnostic precision, predicting disease trajectory, and facilitating individualised care [7,8].

Artificial intelligence (AI), encompassing machine learning (ML) and deep learning (DL), presents a transformative opportunity to address these challenges. ML refers to algorithms that learn patterns from structured data to generate predictions without explicit human instruction, while DL is a specific form of ML employing multi-layered neural networks capable of autonomously extracting hierarchical features from complex, high-dimensional data such as medical images and genomic sequences [9]. By enabling rapid and systematic analysis of large, complex, and multidimensional datasets, AI facilitates earlier and more accurate diagnoses, improves prognostication, guides objective wound assessment, and advances personalized treatment planning [10,11]. In dermatology, AI has demonstrated strong performance in image-based diagnosis, digital pathology, and prognostic modelling; in genomics, it has substantially improved variant prioritization, splicing prediction, and disease risk stratification [9,12]. While previous reviews have examined AI applications in dermatology and autoimmune blistering disorders broadly, a comprehensive synthesis specifically focused on EB has remained lacking [12].

This structured narrative review examines current evidence on AI applications across the full spectrum of EB care, encompassing genetic diagnosis, wound monitoring, inflammatory endotyping, drug repurposing, and advanced therapeutic design. Emerging evidence on AI-enabled smart wound dressing technologies and three-dimensional (3D) bioprinting as innovative approaches to EB wound management is further integrated, alongside a delineation of key challenges and future directions for clinically responsible AI implementation in this rare disease context.

## 2. Methods

A structured narrative review was conducted to examine current evidence and emerging future directions on AI applications in inherited EB. A literature search was performed across PubMed, Europe PMC, and Web of Science from January 2010 to April 2026 using the following search terms in combination: “epidermolysis bullosa” AND (“artificial intelligence” OR “machine learning” OR “deep learning” OR “neural network” OR “bioprinting” OR “biosensor” OR “drug repurposing” OR “wound monitoring” OR “bioinformatics” OR “transcriptomics”). Only English-language publications were included. Grey literature sources including ClinicalTrials.gov and DEBRA International reports were additionally hand-searched to identify registered clinical trials and ongoing research initiatives. Epidermolysis bullosa acquisita was explicitly excluded as an autoimmune disorder pathophysiologically and clinically distinct from inherited EB.

**Figure 1:**
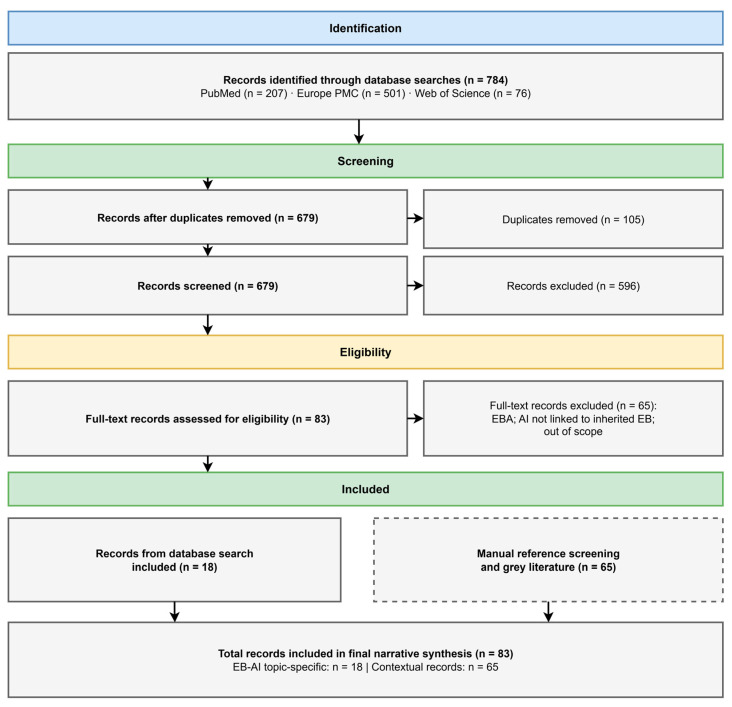
Flowchart of the selection process of records included in this structured narrative review. Artificial intelligence (AI), Epidermolysis bullosa (EB), Epidermolysis bullosa acquisita (EBA).

A total of 784 records were identified across all databases; following removal of 105 duplicate records, 679 unique records were screened by title and abstract for relevance to AI applications in inherited EB. Records were included if they reported AI applications in the diagnosis, monitoring, or treatment of inherited EB, or described computational approaches directly informing EB therapeutic development. Eighteen EB-AI topic-specific records met inclusion criteria and formed the primary narrative synthesis. An additional 65 records addressing EB disease context, AI methodology and frameworks, comparator evidence from analogous rare diseases and chronic wound conditions, and regulatory and ethical frameworks were incorporated through manual reference list screening to contextualise the primary findings, yielding a total of 83 records in the final manuscript. The record selection process is summarised in Figure 1.

**Figure 2:**
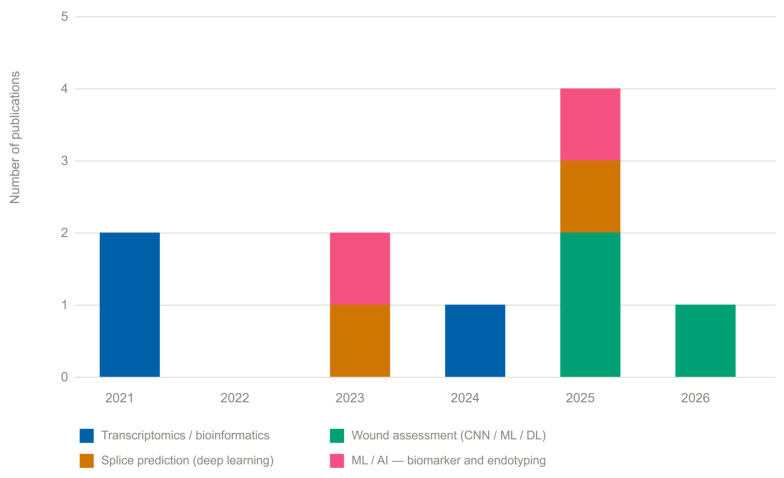
Annual distribution of published AI studies in inherited epidermolysis bullosa identified through the structured literature search (2021–2026), stratified by AI method category. No EB-specific AI publications were identified prior to 2021. Two registered clinical trials currently ongoing are not included (NCT03269474; NCT05843994). Publications include peer-reviewed articles, conference poster presentations, and preprints. Convolutional neural network (CNN), Deep learning (DL), Machine learning (ML).

The temporal distribution of EB-AI topic-specific records identified through the search is summarised in Figure 2, demonstrating a progressive acceleration in published AI research in inherited EB from 2023 onwards, alongside a diversification of applied AI methodologies across genetic, wound assessment, and inflammatory domains.

## 3. Current Applications of AI in EB

The application of AI in EB is an emerging but rapidly evolving field, spanning diagnostic, prognostic, and therapeutic domains. A summary of the principal AI methodologies and their respective applications across the spectrum of EB care is provided in Table 1. The following sections examine each of these domains in detail.

### 3.1. AI Assisted Genetic Diagnosis of EB

Genomic analysis using next-generation sequencing (NGS), whole-exome sequencing (WES), or whole-genome sequencing (WGS) generates massive multidimensional datasets required to identify causative mutations in EB genes [24,25]. The scale and complexity of these datasets often exceed the capacity of traditional analytical methods, making reliable identification of pathogenic variants challenging, particularly given the broad allelic heterogeneity, extensive variant of uncertain significance (VUS) burden, and significant phenotypic overlap between EB subtypes [26]. Recent advances in ML and DL are increasingly utilized to address these limitations, enabling rapid genomic analysis, enhancing variant prioritization, improving detection of disease-causing mutations, and supporting more accurate genomic interpretation through advanced pattern recognition and predictive modeling [27,28,29,30]. The integration of AI-driven genomic tools into EB diagnostic workflows has the potential to substantially reduce the diagnostic latency, often spanning months to years, that many affected individuals and families experience [4].

#### 3.1.1. Comprehensive Genome Interpretation and Variant Prioritization

Notable AI frameworks, such as Fabric GEM, act as an electronic clinical decision support systems (eCDSS) that expedites genome interpretation in rare genetic diseases [31]. Fabric GEM integrates comprehensive genomic data, including structural variants (SVs), single-nucleotide variants (SNVs), and insertions/deletions (Indels), with phenotypic descriptions using Human Phenotype Ontology (HPO) terms and applies Bayesian methods to score and rank candidate genes and diagnoses [27,31]. By aligning patient-specific EB-associated features such as skin fragility, blistering pattern, mucosal involvement, nail dystrophy, or scarring with curated gene-disease relationships, this approach can assist in prioritizing likely pathogenic variants across the major EB genes [1,3]. Although formal evaluation in EB cohorts is awaited, validated performance in broader rare genetic disease cohorts supports its applicability in this setting [31,32]. Complementing genome-level interpretation, ensemble ML methods including REVEL and MVP augment variant pathogenicity prediction by integrating multiple functional annotation features derived from evolutionary conservation, biochemical properties, and structural data, demonstrating improved performance over individual predictors in rare missense variant classification [28,29,30]. Deep neural network approaches such as PrimateAI and CADD further expand the repertoire of computational tools potentially applicable to EB variant interpretation [27,30]. Together, these tools are transforming the genomic diagnostic pipeline from a labor-intensive manual process toward an automated, prioritized, and clinically actionable workflow.

#### 3.1.2. AI-Driven Splicing Prediction and Genotype-Phenotype Correlation

Deep learning has enabled substantially more accurate prediction of the functional consequences of variants affecting RNA splicing, a critical and frequently underappreciated mechanism in EB pathogenesis [13]. SpliceAI, a convolutional neural network (CNN) trained on large-scale genomic datasets, predicts the impact of genetic variants on RNA splicing by analyzing 10,000 nucleotides of genomic context around a variant of interest, assigning a probability score reflecting the likelihood that the variant disrupts or creates splice sites [33]. In an independent validation study evaluating 285 NF1 variants, SpliceAI achieved a sensitivity of 94.5% and specificity of 94.3% at a Δ score threshold of >0.22, with an area under the receiver operating characteristic curve (AUC-ROC) of 0.975, outperforming conventional splice prediction tools [33]. In EB, SpliceAI has been applied to characterize aberrant transcripts in JEB, particularly in genes encoding laminin subunits (LAMB3, LAMC2) and COL17A1 [13]. In a JEB cohort of 17 individuals, SpliceAI analysis of five LAMB3 splice site variants correctly predicted out-of-frame and in-frame transcript outcomes that correlated with severe and intermediate phenotypes respectively, with predictions confirmed by RT-PCR and immunofluorescence mapping in evaluated cases [13]. The tool predicts whether aberrant splicing generates out-of-frame transcripts introducing premature termination codons (PTCs), which generally produce nonfunctional protein and correlate with severe JEB phenotypes, or in-frame transcripts preserving partial protein function, which typically correlate with milder presentations [13,34]. This relationship between splicing efficiency and phenotypic severity has been directly quantified: in two siblings compound heterozygous for identical LAMB3 mutations, full-length transcripts harbouring the p.Glu210Lys missense variant were significantly more frequent in the less severely affected sibling (14 of 108 versus 5 of 106 clones; *p* = 0.03), establishing differential splicing as a measurable modifier of JEB phenotype [34]. This capacity to predict phenotypic severity from genotype has direct clinical utility, informing prognosis, genetic counseling, and the selection of mutation-targeted therapeutic strategies.

Furthermore, Pangolin represents an additional DL splicing prediction framework employing neural networks trained on extensive RNA sequencing datasets, facilitating prediction of splice donor and acceptor site gain or loss and variations in exon inclusion [35]. Pangolin has demonstrated superior performance to SpliceAI in splice site prediction, achieving a mean top-1 accuracy of 79% and AUPRC of 0.85 across tissues, compared with 75% and 0.77 respectively for SpliceAI, with a Spearman correlation of 0.79 between predicted and experimentally measured splicing effects of individual nucleotide variants [14]. In RDEB, concordant SpliceAI and Pangolin predictions for two novel deep intronic COL7A1 variants characterised pathogenic splice site disruption, enabling identification of variants that would have been missed by standard diagnostic pipelines, with quantified Δ scores detailed in Table 1 [14]. The combined application of complementary DL splicing tools thus enables a more comprehensive and clinically actionable characterization of the EB variant landscape, from common exonic mutations to rare and challenging deep intronic pathogenic variants. Current evidence positions DL-based splicing prediction as a preclinical in silico tool with retrospective validation in patient-derived samples; prospective integration into routine EB genomic diagnostic workflows, with RNA-level confirmatory testing of high-confidence predictions, represents the next translational priority.

### 3.2. AI in EB Wounds Monitoring

Accurate and longitudinal wound monitoring in EB is essential for assessing disease severity, tracking progression, and evaluating treatment response [36]. Conventional approaches, including serial clinical examination, standardized photography, and validated scoring instruments such as the Epidermolysis Bullosa Disease Activity and Scarring Index (EBDASI) and the Birmingham Epidermolysis Bullosa Severity Score (BEBS), have improved standardization but remain limited by interobserver variability, significant time burden, and patient tolerability concerns [37,38]. Repeated detailed skin examinations are poorly tolerated by patients with extensive painful wounds, constraining the feasibility of frequent longitudinal monitoring [39,40]. AI-powered imaging and digital platforms directly address these limitations, enabling objective, reproducible, scalable, and patient-friendly wound assessment [9].

#### 3.2.1. Automated Lesion Segmentation and Remote Monitoring

AI enables remote and continuous evaluation of EB lesions, substantially reducing the burden of frequent in-person clinical visits, a significant benefit for patients who often live far from specialist centers and for whom travel itself carries risk of skin trauma [41]. Mobile and web-based platforms integrating ML algorithms, such as Tissue Analytics, allow patients or caregivers to upload standardized serial wound photographs for automated analysis and longitudinal tracking of wound parameters including area, tissue composition, and healing trajectory [16]. This approach aligns with patient-centered care models and decentralized clinical trial designs that maintain high-quality outcome data while minimizing participant burden [42].

Scarletred^®^ Vision, a European Conformity (CE)-marked validated medical imaging device, employs CNNs combined with a signal-calibrated skin reference patch to standardize image acquisition across variable lighting and camera conditions, enabling automated segmentation of EB lesions, isolation of affected from healthy tissue, and precise calculation of affected body surface area [15]. The platform was evaluated in a proof-of-concept study using 157 images from 29 patients with EB, demonstrating blister counting with morphological classification (intact, ruptured, or crusted) and tissue type classification across four categories: erythema, healthy skin, crust, and open wound [15]. No formal AUC-ROC or sensitivity metrics were reported at this stage; prospective clinical validation in EB-specific cohorts remains pending. The platform is currently classified as investigational for EB wound assessment purposes despite its CE-marked status for broader dermatological indications [15].

Beyond wound area quantification, AI systems can automatically detect, count, and categorize individual blisters by morphology and can precisely map wound parameters including depth, topology, edge characteristics, and tissue type proportions including granulation tissue, slough, eschar, and healthy epithelium [15,43]. These granular quantitative outputs enable a level of monitoring objectivity and reproducibility that manual clinical assessment cannot consistently achieve.

Objective wound quantification, while necessary, remains insufficient for clinical reasoning without the capacity to situate individual wound profiles within the broader phenotypic spectrum of RDEB [17]. To address this representational deficit, TriDerm was developed as a triplet-based DL framework that derives interpretable wound embeddings from small RDEB cohorts through concurrent integration of wound photography, expert-delineated lesion masks, and structured clinical text [17]. Applied to 53 wound images from 21 RDEB patients, TriDerm combined a DermLIP Vision Transformer backbone adapted via wound-level attention pooling and non-contrastive VICReg learning for the visual modality with soft ordinal embeddings derived from LLM triplet judgements for the textual modality, achieving a balanced expert agreement of 73.5% and Cohen’s κ = 0.446 on multimodal fusion, outperforming the best unimodal visual baseline by 5.6 percentage points [17]. Nearest-neighbour retrieval demonstrated clinically meaningful embedding structure: superficial granulating lesions clustered separately from deep fibrotic ulcerations, indicating that the embedding space captures wound depth, granulation tissue, and inflammatory state as distinct phenotypic axes. TriDerm currently represents a preclinical proof-of-concept conducted at a single centre; external validation in independent RDEB cohorts is required before the framework can be considered for clinical translation [17]. These findings collectively demonstrate that AI-assisted remote monitoring of EB wounds can yield standardised, quantitatively reproducible longitudinal datasets suitable for prognostic modelling, endpoint refinement, and phenotypic characterisation [15,16,17,42]. However, none of the three tools described in this subsection has yet achieved the level of regulatory validation or independent external replication required for routine clinical use; each is appropriately classified as investigational or preclinical at this stage.

#### 3.2.2. AI Driven Prediction of Wound Healing Trajectories

Beyond cross-sectional wound characterization, AI-driven models enable longitudinal prediction of wound healing outcomes, a capability with direct therapeutic implications in EB [43]. These models analyze time-series image data to extract quantitative features including wound area reduction velocity, epithelialization rate, tissue composition trajectory changes over time, erythema intensity dynamics, and surface texture patterns, features that individually and in combination predict wound healing outcomes with increasing accuracy as longitudinal datasets expand [41,43,44]. Such multimodal predictive models enable stratification of wounds by their predicted healing response, supporting clinicians in early identification of non-responders to current therapies and facilitating timely therapeutic modification before irreversible wound complications develop [43,45].

These predictive approaches have been rigorously validated in chronic wound populations including diabetic foot ulcers and pressure injuries [43,44]. In RDEB specifically, a prospective natural history study applied an ML-based wound measurement platform to serial mobile application photographs from 13 participants, comprising 69 wounds and 734 photographs over up to six months (Table 1) [16]. Automated wound area measurements demonstrated high correlation with manual tracing (r = 0.82, *p* < 0.001), and the study established that chronic open wounds, defined as wounds present for more than 12 weeks, were significantly larger at baseline, and substantially less likely to achieve spontaneous closure, and associated with greater pain burden than recurrent wound types [16]. Wound type classification emerged as the strongest independent predictor of spontaneous wound closure, demonstrating that differentiation between chronic open and recurrent wound types should be incorporated as a primary stratification variable in interventional RDEB clinical trial design [16]. The platform is currently investigational for regulatory purposes in the context of RDEB clinical trials; formal endpoint qualification has not been completed.

As EB-specific longitudinal wound datasets expand, a minimum AI endpoint set for EB wound assessment can be proposed. Wound area, measured from standardised photography at weekly intervals, should serve as the primary output, with percentage wound area change per week reported as a continuous healing trajectory rather than a binary closure event [16,43,44]. Wound type should be prospectively classified as chronic open or recurrent over the validated 12-week threshold and used as the primary stratification variable [16]. Wound durability, defined as sustained closure beyond the natural RDEB reopening interval, should be reported alongside time to first closure, since single-timepoint measurements underestimate therapeutic benefit in a disease characterised by recurrent reopening [46]. Secondary outputs can include tissue composition proportions (granulation tissue, slough, fibrin, epithelium, and eschar as percentage of wound bed area) and wound edge characteristics (attached, non-attached, or rolled), derivable from validated deep learning segmentation models [17,41,43]. All imaging-derived endpoints should be consistently paired with validated patient-reported pain and itch scores at identical weekly time points, given the established correlation between wound size trajectory and symptom burden in RDEB [16].

### 3.3. AI in Cutaneous SCC Surveillance in EB

Patients with severe RDEB face a markedly elevated lifetime risk of Cutaneous SCC (cSCC), with tumours typically first emerging in adolescence within areas of chronic ulceration, often displaying subtle early clinical features that are difficult to distinguish from non-malignant wound changes [47]. Accordingly, consensus guidelines recommend intensified surveillance and a low threshold for biopsy of suspicious or recalcitrant wounds, yet the high wound burden in RDEB makes biopsy burdensome [48]. AI-driven cutaneous malignancy detection offers a suite of complementary diagnostic approaches, encompassing image-based tumour recognition, dermoscopic lesion classification, and molecular biomarker integration, that collectively augment clinical surveillance capacity [49,50].

The most clinically proximate AI initiative specific to RDEB-cSCC is an active prospective observational study (NCT05843994) at Northwestern University, enrolling patients with RDEB and a confirmed history of cSCC, from whom standardized high-resolution wound photographs annotated by dermatology and dermatopathology experts are collected [18]. The model is currently in its training phase, with no performance metrics yet published, and is intended for incorporation into a patient- and physician-facing web application enabling home-initiated cSCC triage, specifically targeting patients with limited specialist access and those with procedural reluctance toward repeated invasive skin biopsies [18]. Prospective validation of this tool holds direct implications for diagnostic latency reduction, biopsy rationalisation, and early cSCC detection in this high-risk population [18].

In parallel, the molecular substrate for non-invasive AI-based cSCC surveillance in RDEB has been advanced through a preclinical study exploiting the Micro-RNAs (miRNAs) expression similarity between RDEB-cSCC and head and neck SCC (HN-SCC) to overcome the sample scarcity inherent to rare disease research [19]. The authors leveraged The Cancer Genome Atlas (TCGA) HN-SCC miRNA sequencing data to train an elastic net logistic regression model on miRNA profiles derived from RDEB primary cells, with predictive performance subsequently evaluated on independent HN-SCC, RDEB cell-based, and RDEB exosome datasets [19]. The full 33-feature model achieved AUC-ROC of 99.98% on the HN-SCC test set, 100% on RDEB cell-based data, and 100% on RDEB exosome-derived samples; a simplified three-feature model retained AUC-ROC values of 96.37%, 90.74%, and 100% across the same datasets, respectively (Table 1) [19]. The detection of tumour-associated miRNA signatures within cell-derived exosomes supports the feasibility of non-invasive liquid biopsy approaches for cSCC detection prior to the emergence of clinically overt malignant morphology [19]. Both studies remain at preclinical or trial training stage; prospective liquid biopsy validation in clinical cohorts is required before either approach can be considered for deployment [18,19].

### 3.4. AI-Assisted Analysis of Inflammatory Endotype Profiles

Patients with RDEB experience a systemic hyperinflammatory disorder that extends well beyond cutaneous manifestations, with chronic systemic inflammation contributing to impaired wound healing, nutritional compromise, anemia, and SCC development [20,51]. A systematic review and meta-analysis of tissue and systemic inflammation in DEB confirmed that inflammatory dysregulation is a consistent and measurable feature across disease subtypes, underscoring the need for objective, scalable tools capable of quantifying and stratifying this inflammatory burden [51]. AI prediction models are being applied to characterize complex systemic biological data, or endotypes, that drive this disease progression beyond cutaneous symptoms [20]. A systems immunology approach integrating cytokine levels, eicosanoid profiles, lipid mediators, and immune cell subset counts into an integrated inflammation-immunity score (IIS), derived using a random forest classifier, has been applied to a single-centre cohort of 12 non-end-stage RDEB adults (8 females, 4 males; mean age 34.6 ± 8.8 years; IscorEB mean 78 ± 25) compared with 9 age-matched healthy controls [20]. Model performance was evaluated by receiver operating characteristic (ROC) curves generated from internal 70/30 training/validation splits of the same cohort, repeated across 50 iterations; no independent external validation cohort was used [20]. Although the IIS model did not predict overall clinical severity scores or total wound area, it successfully identified elevated eicosanoid inflammatory markers like thromboxane B2 (TXB2) and prostaglandin E2 (PGE2), as reliable computational predictions with AUC-ROC values of 0.94 ± 0.12 and 0.98 ± 0.06 respectively (Table 1), confirming the hyperinflammatory systemic phenotype of RDEB and highlighting prostanoid signalling pathways as potential therapeutic targets [20]. AI prediction models and principal component analysis further demonstrated that RDEB endotypes are not solely confined to cutaneous pathology but reflect complex systemic immune dysregulation, characterised by activated and effector T cell populations, dysfunctional natural killer cell signatures, and a pro-inflammatory lipid profile [20]. These findings reposition RDEB conceptually from a structurally defined skin disorder to a systemic inflammatory disease with discrete, computationally characterised immune subtypes amenable to targeted intervention [51].

The integration of multi-omics datasets spanning transcriptomics, proteomics, lipidomics, and immune cell profiling with AI-powered endotype classification holds significant promise for biologically informed patient stratification, enabling selection of anti-inflammatory interventions matched to individual inflammatory profiles rather than applying uniform treatment protocols to a phenotypically heterogeneous population [20,51]. Monitoring these markers enhances clinician understanding of the hyperinflammatory state that perpetuates chronic wounding and drives SCC development, while providing the structured longitudinal datasets that future ML-based disease activity models require [20,51]. The direct application of AI to inflammatory endotype characterisation in RDEB currently rests on this single proof-of-concept cohort, exclusively comprising adults with severe RDEB, with internal cross-validation only; generalisability to other EB subtypes, and paediatric populations remains undemonstrated. Prospective multi-institutional data-sharing initiatives generating larger, genotypically diverse datasets will be essential before ML-based endotype classification can be meaningfully integrated into clinical practice [20].

## 4. Challenges and Limitations

Despite the substantial promise documented across these application domains, translation of AI into routine clinical practice for rare genodermatoses such as EB remains constrained by fundamental methodological, clinical, and ethical barriers [52].

AI systems require large, well-annotated datasets for robust training, validation, and generalizability. The rarity of EB, with a global prevalence estimated at fewer than 500,000 individuals worldwide, and its marked phenotypic heterogeneity across subtypes, disease severity, age groups, and stages of wound evolution significantly limit data availability [9,12,53]. Small training cohorts increase the risk of overfitting, where models learn dataset-specific noise rather than generalizable disease features, reducing performance when applied to unseen clinical populations [9]. Experiences from AI applications in other rare diseases, including Fabry disease, consistently demonstrate that fragmented datasets, inconsistent phenotyping standards, and limited multicenter collaboration are major barriers to successful model development and deployment challenges that are directly applicable to EB [54]. A critical and currently unmet requirement across all twelve EB-specific AI studies identified is prospective external validation against independent clinical cohorts [14,15,16,17,18,19,20,21,22,23,34,55]. None reported prospective performance on an independent external validation cohort; where models were adapted from other disease domains, such as the fine-tuning of a psoriasis segmentation model for EB lesion delineation [15], transferability was assumed rather than formally quantified against an independent EB dataset. Model credibility cannot be established from internal validation metrics alone; concordance indices, AUC-ROC values, and segmentation agreement scores reported within the same dataset on which a model was trained or tuned are susceptible to optimistic bias and do not predict generalisable performance [9,12]. In rare disease AI, where training cohorts are inherently small, the gap between internal and external performance is typically larger than in common disease applications, and this gap has direct implications for clinical trustworthiness [54]. Future EB AI studies should prospectively report performance on held-out external cohorts, provide calibration curves alongside discrimination metrics, and specify the minimum dataset size and phenotypic diversity required before a model is considered sufficiently validated for clinical use.

Algorithmic bias constitutes an additional critical concern with direct equity implications. Dermatology AI models trained predominantly on datasets from lighter-skinned populations have demonstrated significantly reduced diagnostic performance in underrepresented skin tones and demographic groups in multiple independent validation studies [56,57]. This limitation is particularly relevant to EB, where the visual appearance of erythema, blistering, erosions, and chronic wounds varies across skin phototypes and where marked genotype-phenotype heterogeneity may further reduce model accuracy in underrepresented populations, potentially leading to misclassification, inaccurate severity assessment, and inequitable clinical decision support precisely in those patient communities that already face the greatest barriers to specialist care [1,58,59]. In EB specifically, domain shift is likely to manifest across several axes beyond skin phototype. Wound appearance varies systematically with EB subtype, anatomical location, patient age, and wound chronicity [1], meaning that models trained predominantly on adult RDEB wound images may underperform when applied to junctional EB, dominant DEB, or paediatric cohorts. Standardised photography protocols, including consistent lighting, fixed camera distance, and validated calibration markers, are prerequisites for phototype-stratified model performance reporting [16]. Mitigation strategies should include phototype-stratified subgroup reporting using the Fitzpatrick scale, post-deployment calibration monitoring to detect performance drift over time, and prospective model auditing at defined intervals following clinical deployment [56,57].

The interpretability challenge, the ‘black box’ nature of many DL systems undermines clinician trust, limits accountability in clinical decision-making, and presents a significant barrier to regulatory approval and clinical adoption [9,12,52]. In rare disease contexts such as EB, where clinical decisions may have major life-altering consequences and where affected individuals and families are often highly informed advocates for their own care, the ability to explain and audit AI recommendations is not merely a technical preference but an ethical and regulatory imperative [60,61]. Regulatory frameworks for AI as a medical device are evolving rapidly, both the United States Food and Drug Administration (FDA) and the European Medicines Agency (EMA) are developing specific guidance for AI-based clinical decision support tools, with common principles now established covering evidence generation, lifecycle governance, and human oversight [62]. However, the traditional paradigm of medical device regulation was not designed for adaptive, continuously learning AI technologies, and the approval pathway for such systems remains complex [56]. This challenge is amplified in rare disease contexts, where AI applications face unique vulnerabilities from small cohort sizes, inconsistent phenotyping, and fragmented data, and where comprehensive, harmonised regulatory guidance remains limited, creating uncertainty around validation, documentation, and ongoing performance monitoring requirements [57]. The regulatory pathway for AI tools in EB can be further clarified by explicitly stratifying proposed applications by clinical risk class. Low-risk measurement aids, such as automated wound area tracking and blister quantification, function as software-assisted clinical measurements with direct clinician oversight and require demonstration of analytical validity and reproducibility rather than clinical outcome data for regulatory clearance. Moderate-risk decision support tools, such as wound healing trajectory prediction and inflammatory endotype classification, influence clinical management decisions and require prospective validation against patient outcomes, bias auditing across demographic subgroups, and transparency of model architecture. High-risk applications, including infection prediction, squamous cell carcinoma risk stratification, and treatment escalation prompts for surgical excision or systemic antibiotics, carry direct therapeutic consequence and require randomised or prospective controlled evidence of clinical benefit, explicit human oversight requirements, and post-market surveillance protocols before deployment in EB clinical practice [56,62]. Applying this stratification framework prospectively to EB AI development would align regulatory preparation with the level of clinical risk incurred, reducing the likelihood of premature deployment of high-risk tools and accelerating the pathway for low-risk measurement aids that are already technically mature.

Ethical challenges including data privacy, informed consent for data use in model training, algorithmic accountability, and governance frameworks for AI deployment in pediatric populations further complicate implementation and must be proactively addressed through inclusive stakeholder engagement involving patients, clinicians, ethicists, and regulatory authorities [63,64]. Collectively, these challenges underscore that while AI holds conceptual promise for improving care of EB, substantial scientific, ethical, and infrastructural barriers must be addressed.

## 5. Opportunities and Future Directions

The integration of AI presents a transformative opportunity to advance precision medicine in EB by enabling the synthesis of complex genetic, molecular, imaging, and longitudinal clinical data at a scale and resolution that exceeds the capacity of conventional analytical approaches [52]. Deep neural networks designed to predict the functional consequences of genetic variants including the rapidly expanding class of VUS that constitute a major diagnostic and counselling challenge in EB, may substantially improve diagnostic accuracy and enable genotype-informed personalised care planning for newly diagnosed patients [17]. The expanding landscape of curated genetic and clinical databases for rare dermatologic diseases, including EB-specific registries developed through international collaborative networks, provides an unprecedented opportunity for ML applications to facilitate large-scale pattern recognition across molecular, phenotypic, and longitudinal clinical domains [65]. Emerging genotype-phenotype correlation studies in EB have demonstrated strong, reproducible associations between specific genetic variants and disease severity outcomes, establishing a data foundation for AI-driven predictive models capable of risk stratification, prognostication, and evidence-based early therapeutic decision-making at the individual patient level [24,29]. Advances in AI-powered imaging, digital clinimetry, and automated wound analysis offer objective and reproducible tools for disease monitoring, clinical trial endpoints, and prediction of wound healing trajectories, addressing longstanding challenges in EB outcome assessment [41,45]. The feasibility of mobile health platforms and home-based photography combined with AI analytics further supports decentralized monitoring and natural history studies in EB [16]. Integration of systems immunology data may further enable AI-driven endotype classification, supporting biologically informed patient stratification and targeted anti-inflammatory interventions [20]. Lessons from AI applications in other rare diseases emphasize the importance of augmented intelligence frameworks that enhance clinician expertise, while emerging trends in medical AI highlight the need for explainable, multimodal, and prospectively validated systems to ensure clinical relevance and equity [10,54]. Collectively, these converging developments position AI as a critical enabler of translational research and individualized care in EB, with the greatest near-term impact anticipated across three emerging technology domains that remain prospectively unevaluated in this population: AI-integrated smart wound dressing systems, AI-augmented three-dimensional bioprinting, and AI-driven drug repurposing.

### 5.1. AI-Integrated Smart Wound Dressings and Biosensor Technologies in EB Wound Care

Wound management is the cornerstone of EB care, yet daily dressing changes remain labor-intensive and time-consuming, requiring specialized caregiver support and pharmacological analgesia to manage procedural pain [36,60]. This sustained burden on patients and families emphasizes the urgent need for innovative, technologically advanced wound care solutions [4,6]. Chronic wounds in EB are characterized by a complex, dynamic microenvironment marked by persistent inflammation, fluctuating infection risk, impaired angiogenesis, and repeated cycles of re-injury during dressing changes, rendering passive wound dressings suboptimal [36,66,67]. The emergence of AI-integrated smart wound dressing systems incorporating embedded biosensors capable of continuous, real-time physiological monitoring represents a technological convergence with profound implications for the management of chronic wounds in EB, one that has not yet been formally investigated in this population despite a rapidly maturing evidence base in analogous chronic wound contexts [68,69].

Next-generation smart dressings employ biocompatible polymer matrices, including electrospun nanofibre scaffolds, functionalised with multiparameter sensing elements capable of simultaneously monitoring wound pH, temperature, moisture content, oxygen saturation, and inflammatory biomarker concentrations in situ [68,69]. The biological rationale for this approach in EB is compelling: pH shifts reliably distinguish healing from stagnant or infected wound states; localised temperature elevation constitutes an early, sensitive indicator of nascent infection preceding systemic signs; and tissue oxygen saturation serves as a real time surrogate of wound perfusion [68,69]. Wearable biosensors configured to detect infection associated metabolites, including trimethylamine and uric acid, extend this capability toward non-invasive identification of wound colonisation and biofilm formation, a recurrent and clinically consequential complication in RDEB [70].

The integration of AI into sensor enabled dressing systems transforms continuous physiological data into clinically actionable outputs through several mechanisms. Individualised wound trajectory predictions can be generated by DL algorithms trained on longitudinal multiparameter datasets, these models effectively distinguish between wounds progressing toward healing from those at risk of deterioration, enabling detection of clinical deviations in near real time [68,69]. Closed loop architectures, in which sensor derived signals autonomously modulate dressing drug release profiles, such as upregulating antimicrobial delivery in response to biosensor detected pH elevation or bacterial metabolite accumulation, represent a particularly transformative application for EB, enabling responsive therapeutic delivery at the wound interface without necessitating dressing removal [68,69]. Longer-term integration of wound fluid multiomics data into AI models holds further promise for precision wound care calibrated to each patient’s inflammatory biology, a degree of individualisation unattainable with current passive dressings or episodic clinical assessment [68].

Translation to EB may present a disease-specific engineering challenge that distinguish this application from analogous development in diabetic foot ulcers or pressure injuries. Extreme skin fragility imposes strict limits on sensor application and removal forces, which must remain below the threshold that would itself precipitate blistering and must not compromise the atraumatic properties of EB dressings; this likely necessitates integration into EB-friendly dressing materials, such as soft silicone foam or polymeric membrane dressings, embedding sensor elements within these existing non-adherent layers rather than introducing separate adhesive components [4,6,36]. Sensor placement must accommodate the distribution, irregular morphology, and the substantial heterogeneity of EB wounds, which frequently span flexural surfaces, digits, and curved body contours where rigid or planar sensor arrays would generate localised pressure points; conformable, low-profile designs compatible with multilayer EB dressing regimens are therefore a prerequisite rather than a refinement [4,6,36,66]. Given that EB wound care is delivered either by family caregivers at home or by visiting nurses, often without specialised training in sensor-based technologies, successful deployment will require structured education programmes covering sensor application, troubleshooting, and interpretation of device outputs, delivered through formats that accommodate the considerable existing burden of daily dressing changes [4,6,36,60]. Data transmission architectures must function reliably within home settings of variable connectivity, favouring low-power, intermittent-sync designs over continuous high-bandwidth streaming, with local data buffering to prevent loss of clinically relevant readings [68,69]. Finally, alert thresholds for parameters such as pH shift, temperature elevation, or biomarker accumulation must be calibrated to the elevated baseline inflammatory state characteristic of chronic EB wounds [36], since thresholds derived from acute or non-EB chronic wound populations would likely generate excessive false-positive alerts; iterative threshold calibration against EB-specific longitudinal data, combined with tiered alerting that distinguishes urgent from trend-level signals, will be necessary to avoid alarm fatigue and preserve caregiver trust in the system [68,69]. Meaningful progress will require prospective collaboration between smart materials scientists, EB clinical specialists, and patient advocacy organisations to co-design systems that are mechanically compatible, clinically interpretable, and acceptable to a population for whom every wound-related procedure carries inherent risk of pain and tissue trauma [4,6,60].

### 5.2. Three-Dimensional Bioprinting as a Therapeutic Approach in EB

The 3D bioprinting represents a promising and innovative therapeutic approach for EB, particularly RDEB, by enabling the construction of functional, architecturally defined, biocompatible skin substitutes capable of restoring disrupted dermo-epidermal adhesion [71,72]. For EB, the most transformative application of 3D bioprinting lies in its capacity to incorporate genetically corrected cells or therapeutic molecules directly into printed grafts, enabling targeted wound repair and restoration of the structural proteins whose absence or dysfunction underlies disease pathology [71,72]. Printed constructs incorporating autologous cells that have undergone ex vivo gene correction, for example via CRISPR-Cas9 or lentiviral vector delivery of functional COL7A1, could restore type VII collagen expression and anchoring fibril formation within the dermis, directly addressing the molecular defect in RDEB [71]. Two primary bioprinting approaches are under investigation: in situ bioprinting, in which the bioink is deposited directly onto the wound surface and adapts to wound topography; and in vitro bioprinting, in which grafts are fabricated in the laboratory for subsequent surgical transfer to the wound [71]. While in vitro bioprinted grafts allow rigorous quality control and biomechanical characterisation prior to clinical application, in situ bioprinting eliminates the graft integration barrier, though it demands highly portable and clinically deployable printing systems capable of adapting to the irregular topology of chronic RDEB wounds in real time [71].

Despite its significant therapeutic potential, 3D bioprinting for EB remains entirely at the preclinical and investigational stage, with no clinically approved application established in this population to date [71,72]. Key translational barriers persist across multiple domains: scaling bioink manufacturing to address the large, multifocal, and geographically distributed wound burden demands manufacturing infrastructure currently unavailable in most clinical settings; optimising bioink formulations to simultaneously satisfy requirements for printability, mechanical integrity, controlled biodegradation, and spatially accurate cell organisation remains technically demanding; and immunological compatibility constitutes a fundamental obstacle when allogeneic cell sources are employed, given the breadth of HLA haplotype diversity and the compounding immunological vulnerability inherent to severe RDEB [71,72].

Notably, while clinical translation in EB remains pending, AI-integrated 3D bioprinting has already demonstrated feasibility in other wound types, particularly diabetic foot ulcers, where ML-guided bioink optimisation, AI-assisted high-throughput print condition screening, and automated wound topography reconstruction have been applied to generate patient-specific bioprinted constructs with encouraging preclinical and early clinical results [73]. The integration of AI into the bioprinting workflow for EB, encompassing ML-guided bioink formulation, real-time print quality monitoring, and genetically corrected autologous induced pluripotent stem cell (iPSC) incorporation, therefore represents a logical and tractable translational next step, building on proof-of-concept established in these broader wound care applications, while supportive health policy frameworks ensuring equitable global access will be critical to realising its full public health impact [71,72].

Bioprinting’s clinical relevance in EB will ultimately depend not on manufacturing feasibility alone, but on its capacity to improve outcomes that matter most to patients: durable wound closure, reduced pain and itch, fewer dressing changes, lower infection rates, and, over longer follow-up, reduced risk of SCC at chronically wounded sites [15,16,20,46,48]. The earliest plausible clinical study in EB would likely take the form of a small feasibility trial applying in vitro bioprinted autologous grafts, incorporating collagen VII-corrected cells, to a limited number of chronic EB wounds in a small cohort of adult patients with RDEB. Such a trial would require standardised safety monitoring for graft rejection, infection, and immunogenicity. Efficacy would be assessed using objective wound trajectory metrics, specifically wound area change per week and durability of closure, paired with patient-reported pain and itch scores [16,44].

### 5.3. AI for Drug Repurposing and Advanced Therapeutic Design in EB

Despite advances in understanding the molecular pathology of EB, effective disease modifying therapies remain limited [7]. Drug repurposing, the identification of new therapeutic indications for existing drugs beyond their original approved clinical use, offers a particularly valuable strategy in rare diseases such as EB, where the prohibitive cost and timeline of de novo drug development constrain the conventional pharmaceutical pipeline [74]. AI tools encompassing ML and DL offer a transformative capacity to accelerate this process by integrating complex multimodal datasets, including genomics, transcriptomics, protein drug interaction networks, and biomedical data to predict candidate therapeutics capable of modulating disease relevant pathways [74,75].

Within EB, bioinformatics-guided computational drug repurposing has yielded mechanistically grounded candidate therapeutics. Transcriptomic profiling of RDEB wounds, using reverse transcriptomic analysis, identified methotrexate, simvastatin, and IL-17A inhibitors as candidate compounds capable of reversing pathogenic transcriptional profiles towards those of intact skin, implicating dysregulated cytokine–cytokine receptor interactions, Toll-like receptor signaling, and JAK-STAT pathways as tractable pharmacological targets [7,21]. Similarly, transcriptomic repositioning analysis in EBS revealed aberrant mTOR pathway activation, providing a mechanistic rationale for mTOR inhibition as a candidate therapeutic strategy [22]. Building on this computational framework, an ongoing trial is currently employing gene expression profiling of blistered and non-blistered skin across all EBS subtype, with computational analysis to identify repurposable drugs capable of improving wound healing and reducing pain across the EBS spectrum [55].

Molecular modelling has extended computational therapeutic design in EB beyond drug repurposing, enabling identification of deep intronic COL7A1 splice-altering variants not detectable by conventional diagnostic approaches and directly informing the development of antisense oligonucleotide (ASOs)-based splice modulation strategies that have demonstrated correction of aberrant splicing and partial restoration of functional collagen VII transcripts in patient-derived models [23,76]. In addition, molecular modeling has supported the potential use of pharmacological chaperones, small molecules designed to stabilize misfolded or partially functional proteins, as a mutation-specific approach to improving collagen VII folding, trafficking, and stability in patients harbouring missense variants [77]. Collectively, these studies establish a mechanistic and translational foundation upon which AI-augmented approaches can build. The integration of ML models trained on patient-derived EB skin molecular profiles could systematically prioritise druggable targets and rank repurposing candidates by predicted therapeutic relevance and safety profile, substantially extending what reverse transcriptomic approaches alone can achieve [74,75].

However, the clinical value of such computational predictions depends on a structured prioritisation framework rather than reversal score alone. Candidate compounds should be ranked by mechanistic plausibility; the pathways identified through reverse transcriptomic correlation, cytokine-cytokine receptor interactions, Toll-like receptor signaling, JAK-STAT, and mTOR, require independent confirmation of relevance to EB pathophysiology through protein expression studies or functional assays before being considered mechanistically plausible therapeutic targets [21,22]. Paediatric safety and immunomodulatory effects warrant particular attention in EB. Methotrexate, simvastatin, IL-17A inhibitors, and mTOR inhibitors all have established paediatric use in other indications. However, the immunomodulatory effects of these agents, particularly IL-17A and mTOR inhibitors, may impact wound healing despite favourably altering inflammatory transcriptional signatures, and may concurrently compound infection susceptibility in patients already prone to chronic wound colonisation [67]. Future studies should therefore report functional validation in patient-derived models, such as RDEB keratinocyte or fibroblast cultures and iPSCs [23,76], confirming that computationally predicted reversal of pathogenic transcriptional signatures translates into measurable improvements in collagen VII expression, anchoring fibril formation, or inflammatory cytokine secretion. Where candidates progress to clinical evaluation, pragmatic adaptive trial designs incorporating the proposed minimum endpoint set for EB wound assessment would allow efficacy signals to be detected efficiently across the rare and heterogeneous EB population while permitting early termination of candidates that fail to demonstrate clinically meaningful benefit [16,44].

### 5.4. Large Language Models as an Emerging Diagnostic and Monitoring Adjunct in EB

The rapid proliferation of multimodal large language models (LLMs) represents a distinct and rapidly evolving direction with direct relevance to EB care. Unlike purpose-built dermatology AI tools, general-purpose LLMs such as ChatGPT, Gemini, and Claude are freely accessible to the public, and patients, including those with EB and their caregivers, who increasingly photograph their own skin lesions and submit them to these models for informal diagnostic guidance [78,79]. This pattern of unsupervised use, occurring regardless of formal validation, makes the performance characteristics of these models directly relevant to EB care even in the absence of EB-specific studies. Across several recent evaluations, the capability of LLMs to perform four tasks of direct relevance to EB has been assessed: primary disease identification, lesion severity assessment, treatment recommendation, and differentiation from clinical mimickers [78,79,80]. In hidradenitis suppurativa, ChatGPT-4o achieved a diagnostic accuracy of 87.3% from clinical images, with Hurley stage severity assessment accuracies ranging from 65.5% to 82.6% across models, and appropriate treatment recommendations in up to 88.7% of correctly identified cases [80]. In acne and rosacea, GPT-4o demonstrated a sensitivity of 93.0% and specificity of 97.7% for primary diagnosis, though subtype classification accuracy was substantially lower, at 54.6% for acne and 50.0% for rosacea [78]. In a third evaluation focused on basal cell carcinoma and its clinical mimickers, including SCC, melanoma, and benign lesions such as nevi and seborrheic keratosis, ChatGPT-5, Claude Sonnet 4, and Gemini 2.5 Flash achieved detection accuracies of 75%, 64.3%, and 50.7% respectively for clinical images, and 55.2%, 69.8%, and 50.9% for dermoscopic images, with subtype classification accuracy remaining modest across all models and performance declining substantially in crusted and flat lesions [79].

The relevance of this emerging evidence to EB lies primarily in supporting earlier disease recognition rather than in malignancy surveillance. EB shares with hidradenitis suppurativa the problem of substantial diagnostic delay, the condition in which LLMs achieved their highest accuracy in this evidence base, suggesting that image-recognising LLMs could, in principle, serve as an adjunctive aid for earlier recognition of disease onset in settings with limited specialist access [80]. However, the evidence reviewed here was generated in conditions structurally distinct from EB, and no study to date has evaluated LLM performance on EB-specific wound images, blistering lesions, or the distinctive scarring seen in RDEB. Given the consistently poor performance of all evaluated models on crusted and flat lesions, the morphological features that predominate in chronic EB wounds, and the modest positive predictive values observed for malignancy detection [81], LLM-based tools should not be considered for SCC surveillance in EB at present. Any future role for LLMs in EB would therefore be as an adjunctive aid prompting earlier clinician review of new or changing lesions in resource-limited settings, rather than as an autonomous diagnostic or monitoring system, pending EB-specific prospective validation. A summary of the current evidence base, AI integration, translational barriers, and future priorities for each of these emerging technology domains is provided in Table 2.

### 5.5. Translational Priorities and Implementation Framework

To enable clinical implementation, an integrated translational framework is required encompassing several interdependent priorities. The expansion of internationally interoperable disease registries for patients with EB, incorporating standardised multicentre wound imaging protocols and longitudinal multiomics data collection, is foundational for AI model training and external validation at the scale that rare disease populations demand, and represents the single intervention most likely to accelerate AI adoption in EB in the near term, as it underlies external validation across every technology domain discussed in this review [81]. Clinical trial designs in patients with EB must evolve beyond conventional randomised controlled trial frameworks to incorporate AI-derived digital wound assessment endpoints with patient-reported outcomes, as static fixed-endpoint designs are fundamentally unsuited to evaluating the dynamic, continuously updated algorithmic outputs that AI-integrated tools generate, and insufficiently powered to validate such tools within the recruitment constraints inherent to a rare and geographically dispersed population [82]. Emerging regulatory frameworks for AI-enabled medical devices define the prospective validation and approval pathways within which AI-integrated wound monitoring devices, bioprinted constructs, and computational repurposing pipelines must be rigorously evaluated before clinical deployment, with internationally harmonised standards essential to accommodate the globally distributed nature of patients with EB [83]. Ethical governance structures addressing algorithmic bias, data ownership, and equitable access to high-cost regenerative technologies must equally be embedded within this framework from inception rather than retrofitted post-development [63,64].

**Figure 3:**
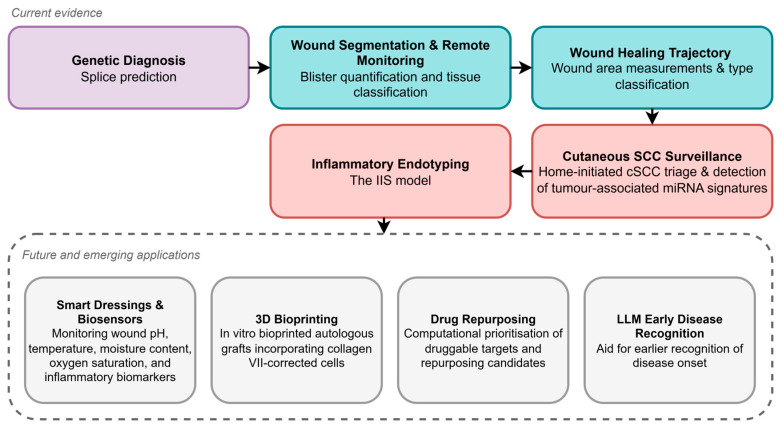
Conceptual workflow of current and future AI applications across the epi-dermolysis bullosa patient pathway. Artificial intelligence (AI), Epidermolysis bullosa (EB), Squamous cell carcinoma (SCC).

Critically, each of these priorities must be co-designed with patients with EB and multidisciplinary care teams to ensure usability within the high-dependency, resource-intensive care environments that characterise severe disease [6]. Collectively, these translational priorities position AI not as a single technology but as an enabling infrastructure across the full therapeutic landscape of EB. The convergence of AI-integrated wound monitoring, 3D bioprinting, and computational drug repurposing offers a plausible route toward patient-specific, precision wound care in EB, provided that technical advances are matched by rigorous clinical validation, proportionate regulation, and implementation strategies grounded in the realities of high-dependency EB care [74]. Figure 3 synthesises the AI applications discussed throughout this review into a single conceptual workflow spanning the EB patient pathway, distinguishing EB-validated evidence from extrapolated or future applications.

## 6. Conclusions

AI holds significant promise for advancing the care of EB by enhancing diagnostic accuracy, treatment planning, and longitudinal disease monitoring through the integration of complex clinical and molecular data [11,52]. Evidence from dermatology and rare disease research supports the use of AI as an augmentative tool that complements clinician expertise rather than replaces it, a model particularly suited to pediatric and genetically heterogenous disorders such as EB [9,10,57]. As AI continues to evolve within medicine, its successful translation into EB care will depend on transparent model design, rigorous clinical validation, and responsible governance to ensure equitable safe and clinically meaningful implementation [46,52,83].

## Figures and Tables

**Table 1:** Overview of AI methods and emerging technologies applied in EB research and clinical practice.

AI Method	EB Application Domain	Study	Tools/Approach	Key Performance Metrics	Clinical Readiness
Splice prediction (deep learning)	Genotype-phenotype correlation in JEB; characterisation of LAMB3, COL17A1 splice variants	Wen et al. (2023) [13]	SpliceAI	Splice site delta scores reported; correct transcript predictions validated by RT-PCR	Preclinical: retrospective in silico validation in patient-derived samples; prospective diagnostic evaluation not yet performed
	Identification of pathogenic deep intronic COL7A1 variants in RDEB; concordant prediction of splice site disruption and exon skipping	Buianova et al. (2025) [14]	SpliceAI and Pangolin	c.6501+6T>C: SpliceAI donor gain Δ = 0.38, Pangolin gain = 0.46; c.7759-3C>G: SpliceAI acceptor loss = 0.92, Pangolin loss = 0.84	
CNN: wound segmentation and tissue classification	Automated EB lesion segmentation, blister counting, open wound detection, and tissue classification	Molnarova et al. (2025) [15]	Scarletred^®^ Vision (CNN; U-Net with EfficientNet)	Proof-of-concept; no formal AUC or sensitivity reported; 157 images from 29 patients	Investigational, CE-marked SaMD platform; prospective clinical validation in EB pending
ML: automated wound area measurement	Remote longitudinal RDEB wound monitoring via mobile application home photography	Fulchand et al. (2025) [16]	Tissue Analytics ML platform	Correlation r = 0.82 (*p* < 0.001) vs. manual ImageJ tracing; 74 wound photographs	Investigational, prospectively applied in RDEB natural history study; regulatory validation pending
Multimodal DL: wound embeddings	Wound similarity retrieval and case-based clinical reasoning in RDEB	Kabus et al. (2026) [17]	TriDerm (DermLIP-ViT-B + GPT-OSS-120B; VICReg + SOE fusion)	Balanced agreement 73.5%; Cohen’s κ = 0.446 (moderate); 53 wound images from 21 RDEB patients	Preclinical: single-centre proof-of-concept preprint; external validation required
ML: clinical triage	AI-assisted cSCC versus chronic wound differentiation in RDEB	NCT05843994 [18]	Patient-facing AI web application	Currently in training phase; no performance metrics published	Clinical trial: ongoing prospective validation; not yet deployed
ML: elastic net logistic regression	miRNA-based non-invasive cSCC liquid biopsy detection in RDEB	Zauner et al. (2023) [19]	Elastic net logistic regression trained on TCGA HN-SCC miRNA data	AUC-ROC: SIG-33 model 99.98% (HN-SCC test), 100% (RDEB cells), 100% (RDEB exosomes); SIG-3 minimal model 96.37%/90.74%/100%	Preclinical: cell and exosome model validation; prospective liquid biopsy trial required
ML/AI: inflammatory endotyping	Characterisation of systemic immune dysregulation and hyperinflammatory endotypes in RDEB adults	Hirt et al. (2025) [20]	Random Forest (IIS); UMAP; PhenoGraph; FlowSOM	AUC: TXB2 prediction 0.94 ± 0.12; PGE2 0.98 ± 0.06; IscorEB 0.17 ± 0.24 (poor); wound area 0.44 ± 0.24 (poor); 12 RDEB adults	Preclinical: single-cohort proof-of-concept; internal 70/30 cross-validation only; independent external validation required
Transcriptomics/bioinformatics	Computational drug repurposing in RDEB wounds; identification of methotrexate, simvastatin	Onoufriadis et al. (2022) [21]	L1000FWD reverse transcriptomics; limma; GAGE; GSVA	>2000 DEGs identified; pathway FDR *p* < 0.01; 6 RDEB patients	Preclinical: in silico only; no prospective clinical validation to date
	Transcriptomic repositioning identifying mTOR pathway activation in EBS; mTOR inhibitor repurposing	Lee et al. (2022) [22]	CLUE Connectivity Map; Ingenuity Pathway Analysis; DESeq2	1276 DEGs; mTOR inhibitor CMap score −90.77; clinical pilot: 2 patients improved after 12 weeks topical sirolimus	Investigational: computational plus open-label pilot (*n* = 2); registered trial NCT03269474 ongoing
	Identification of deep intronic COL7A1 splice variants; ASO-mediated splicing correction	Pironon et al. (2024) [23]	Human Splicing Finder; ESEfinder; ASO splice modulation	Normal splicing restored in patient-derived keratinocytes and fibroblasts; partial C7 restoration demonstrated	Preclinical: patient-derived model validation; no clinical trial yet

Artificial intelligence (AI), Antisense oligonucleotide (ASO), Area under the receiver operating characteristic curve (AUC-ROC), Convolutional neural network (CNN), Cutaneous squamous cell carcinoma (cSCC), Deep learning (DL), Epidermolysis bullosa simplex (EBS), Head and neck squamous cell carcinoma (HN-SCC), Inflammation immunity score (IIS), Junctional epidermolysis bullosa (JEB), Machine learning (ML), MicroRNA (miRNA), Recessive dystrophic epidermolysis bullosa (RDEB), Software as a medical device (SaMD), Soft ordinal embedding (SOE), The Cancer Genome Atlas (TCGA), Type VII collagen (C7).

**Table 2:** Emerging AI-integrated therapeutic technologies in EB: current evidence, translational barriers, and future priorities.

Technology Domain	Current Evidence Base	AI Integration	EB-Specific Translational Barriers	Future Priorities	Clinical Translation Stage
AI-integrated smart wound dressings and biosensor technologies	Proof-of-concept in diabetic foot ulcers, pressure injuries, and chronic wounds; not yet evaluated in EB [68,69].	DL-based wound trajectory prediction; closed-loop autonomous drug release modulated by biosensor-derived signals; real-time multiparameter physiological monitoring	Extreme skin fragility constrains sensor application and removal forces; heterogeneous wound topology demands adaptable sensor architectures; atraumatic compatibility essential	Prospective co-design studies with EB clinical teams and patient advocacy organisations; mechanical biocompatibility testing in EB wound models; regulatory validation as AI-enabled medical devices	Preclinical (non-EB proof-of-concept)
Three-dimensional bioprinting as a therapeutic approach	Proof-of-concept in diabetic foot ulcers; no approved clinical application in EB to date [71,72,73]	ML-guided bioink formulation optimisation; AI-assisted high-throughput print condition screening; automated wound topography reconstruction for patient-specific construct fabrication	Bioink manufacturing scalability; HLA compatibility with allogeneic cell sources; irregular chronic wound topology; absence of clinical-grade portable bioprinting systems	Adaptive trial designs evaluating bioprinted constructs in RDEB; genetically corrected autologous iPSC integration; AI-powered quality monitoring; international manufacturing infrastructure development	Conceptual (extrapolated from non-EB preclinical work)
AI for drug repurposing and advanced therapeutic design	Bioinformatics-guided transcriptomic repurposing identifies methotrexate, simvastatin, IL-17A inhibitors in RDEB; mTOR inhibition in EBS; ongoing clinical trial NCT03269474 [7,21,22,55,74,75]	ML models trained on patient-derived molecular profiles for target prioritisation and candidate ranking; AI extends beyond reverse transcriptomics to multimodal data integration	Limited EB-specific multi-omic training datasets; small patient population constrains model validation; absence of prospective AI-guided repurposing trials in EB	AI-augmented drug-target interaction modelling; integration with expanding EB patient registries; prospective validation of ML-prioritised candidates in adaptive clinical trials	Computational (hypothesis-generating) with one ongoing early-phase clinical trial
Large language models as a diagnostic and monitoring adjunct	Diagnostic accuracy of 87.3% in hidradenitis suppurativa; sensitivity of 93.0% in acne/rosacea; detection accuracies of 50.7–75% for BCC and mimickers, with poor performance in crusted and flat lesions [78,79,80]	Multimodal image-based primary disease identification, severity staging, treatment recommendation, and differentiation from clinical mimickers using freely accessible general-purpose LLMs	Poor performance on crusted and flat lesions, the predominant morphology of chronic EB wounds; no validation on EB-specific blistering, scarring, or wound images; modest positive predictive values raise false-positive concern for malignancy flagging	EB-specific validation studies on blistering and chronic wound images; calibration of alert thresholds to avoid false-positive malignancy flags; positioning as adjunctive aid prompting clinician review rather than autonomous diagnostic tool	Validated in non-EB dermatological conditions only; no EB-specific evaluation to date

Artificial intelligence (AI), Deep Learning (DL), Epidermolysis bullosa simplex (EBS), Human leucocyte antigen (HLA), Induced pluripotent stem cell (iPSC), Machine learning (ML), Recessive dystrophic epidermolysis bullosa (RDEB).

## Data Availability

No new data were created or analyzed in this study. Data sharing is not applicable.

## Abbreviations

EB: Epidermolysis Bullosa. EBS: Epidermolysis Bullosa simplex. JEB: Junctional Epidermolysis Bullosa. DEB: Dystrophic Epidermolysis Bullosa. RDEB: Recessive Dystrophic Epidermolysis Bullosa. SCC: Squamous cell carcinoma. AI: Artificial intelligence. ML: Machine Learning. DL: Deep Learning. 3D: Three-dimensional. CNN: Convolutional neural networks. NGS: Next-generation sequencing. WES: Whole-exome sequencing. WGS: Whole-genome sequencing. VUS: Variant of uncertain significance. eCDSS: Clinical decision support systems. SVs: Structural variants. SNVs: Single-nucleotide variant. Indel: Insertiondeletion. HPO: Human Phenotype Ontology. PTCs: Premature termination codons. EBDSAI: Epidermolysis Bullosa Disease Activity and Scarring Index. BEBS: Birmingham Epidermolysis Bullosa Severity Score. CE: European Conformity. cSCC: Cutaneous squamous cell carcinoma. miRNAs: Micro-RNAs. HN-SCC: Head and neck SCC. TCGA: The Cancer Genome Atlas. AUC-ROC: Area under the receiver operating characteristic curve. IIS: Inflammation immunity score. ROC: Receiver operating characteristic. TXB2: Thromboxane B2. PGE2: Prostaglandin E2. FDA: Food and Drug Administration. EMA: European Medicines Agency. iPSC: Induced pluripotent stem cell. ASO: Antisense oligonucleotide. LLM: Large language models.
